# Addiction Memory, Family Functioning, and Depression in Illicit Drug Users: Self-Esteem as a Mediator

**DOI:** 10.3390/bs14121185

**Published:** 2024-12-12

**Authors:** Li Zeng, Xiaojun Zhou, Yuge Lei, Jiayan Chen

**Affiliations:** 1 School of Public Health, Jiangxi Medical College, Nanchang University, Nanchang 330006, China; 406500220001@email.ncu.edu.cn (L.Z.); zhouxiaojun@ncu.edu.cn (X.Z.); 416500230050@email.ncu.edu.cn (Y.L.); 2 Jiangxi Provincial Key Laboratory of Disease Prevention and Public Health, Nanchang University, Nanchang 330006, China

**Keywords:** substance abuse, addiction memory, family functioning, depression, self-esteem, mediating effect

## Abstract

Depression is a common issue among illicit drug users. However, the interaction between addiction memory, family functioning, and depressive symptoms remains insufficiently examined. This study investigates the relationship between addiction memory, family functioning, and depressive symptoms, with self-esteem serving as a mediator. A random sample of 600 illicit drug users from Hubei, China, was evaluated using the Addiction Memory Intensity Scale (AMIS), Family APGAR Index, Patient Health Questionnaire (PHQ-9), and Rosenberg Self-Esteem Scale (RSES). A factorial algorithm was used to parcel the AMIS, PHQ-9, and RSES items, and structural equation modeling was applied to examine the mediation effect. The model fit the data well (*χ*^2^/*df* = 2.248, CFI = 0.956, NNFI = 0.966, RMSEA = 0.046, SRMR = 0.040), with significant path coefficients (*p* < 0.05). Addiction memory was positively correlated with depression (*β* = 0.195, *p* < 0.001), while family functioning negatively correlated with depression (*β* = −0.113, *p* = 0.008). Both addiction memory and family functioning indirectly influenced depression through self-esteem, with mediating effects equal to 1.154 (95% *CI*: 0.106 to 0.209) and −0.097 (95% *CI*: −0.154 to −0.047). These findings suggest that interventions targeting addiction memory and family functioning may alleviate depressive symptoms by improving self-esteem among illicit drug users.

## 1. Introduction

The World Drug Report 2024 indicates that approximately 292 million people worldwide used drugs in 2022, a 20% increase from a decade ago [1]. Depressive symptoms are often associated with substance abuse, and they are influenced by various factors [2]. For example, the frequency of substance use, stress, and mental health issues can impact depressive symptoms [3,4]. Additionally, family dynamics are key factors affecting depressive symptoms [5]. Therefore, exploring these associations is essential for developing effective interventions and improving treatment outcomes.

Addiction memory, which involves substance-related cues and environments [6], may play a key role in the development of depression. Exposure to substance-related cues or environments can trigger intense cravings driven by long-term addiction memory, leading to continued substance abuse [7]. The Affective Processing Model of Negative Reinforcement suggests that the primary drive for substance abuse is the desire to escape negative emotions [8]. Memories of euphoria associated with substance use heighten sensitivity to related cues. When substance use stops, individuals may experience anxiety, depression, and other negative emotions, raising the risk of relapsing to avoid these distressing feelings [9]. This suggests that addiction memory may contribute to the onset of depression.

Family, as a key source of social support, plays a crucial role in the recovery of substance users. Family functioning refers to the roles a family performs, creating the necessary environment for member development, including communication, emotional responses, and involvement [10]. Low-functioning families can generate negative emotions among members, potentially leading to substance abuse as a means of escaping real-life pressures. Research indicates that individuals with dysfunctional family dynamics are more likely to develop depressive symptoms [11]. Therefore, this study proposes the following hypotheses:

**Hypothesis 1.**  *Addiction memory is positively associated with depression.*

**Hypothesis 2.**  *Family functioning is negatively associated with depression.*

Self-esteem is rooted in self-perception and involves an individual’s evaluation of their worth [12]. Coopersmith identified four prerequisites for self-esteem, all linked to parental interactions, highlighting a close relationship between family and self-esteem [13]. Self-esteem levels largely depend on childhood or adolescent family experiences. Children or adolescents from dysfunctional families often communicate less with their parents, resulting in a diminished sense of self-identity and lower self-esteem [14]. Studies indicate that substance users have lower self-esteem than non-users [15], and individuals with low self-esteem are more likely to engage in risky behaviors, including substance abuse. Numerous studies have demonstrated the impact of self-esteem on depression [16,17]. The vulnerability model posits that individuals with low self-esteem are more likely to reject help and exhibit social avoidance, which further promotes depressive symptoms [18]. Additionally, research indicates that family dysfunction can result in low self-esteem and loneliness, potentially triggering depression [19]. Therefore, this study proposes the following hypotheses:

**Hypothesis 3.**  *Self-esteem mediates the relationship between addiction memory and depression.*

**Hypothesis 4.**  *Self-esteem mediates the relationship between family functioning and depression.*

## 2. Materials and Methods

### 2.1. Participants

This study employed a multi-stage random sampling method. First, all six drug rehabilitation centers in Hubei Province, China, were stratified by gender. One center from each stratum was then randomly selected as the survey unit. Finally, six hundred illicit drug users from the two centers were randomly selected from a staff-provided list. If a selected patient refused to participate, the next eligible patient on the list was chosen. The inclusion criteria were as follows: illicit drug users meeting the diagnostic criteria for substance use disorders given in the Diagnostic and Statistical Manual of Mental Disorders (Fifth Edition) [20]; over 18 years old; and having undergone regular detoxification treatment for more than 15 days, without significant withdrawal symptoms. Exclusion criteria included illiteracy, cognitive impairments, or other mental disorders that interfered with one’s ability to complete the questionnaire. Six hundred questionnaires were distributed, with nine invalid responses removed due to patterned answers. This resulted in 591 valid responses, yielding a response rate of 98.5%. This study was approved by the Research Ethics Committee of Nanchang University—BioMedicine (Approval No. NCUREC202409011). All participants provided informed consent to participate in the survey.

### 2.2. Measures

#### 2.2.1. Addiction Memory Intensity Scale (AMIS)

The AMIS was used to assess the intensity of addiction memory in participants. It contains nine items and is scored using a five-point Likert scale, with higher scores indicating greater intensity of addiction memory [21]. The Cronbach’s alpha for the AMIS was 0.91 in this study.

#### 2.2.2. Family APGAR Index

The APGAR index was used to assess family functioning across five dimensions: Adaptation, Partnership, Growth, Affection, and Resolve. It includes five items and is evaluated using a three-point Likert scale, with higher scores indicating better family functioning [22]. In this study, the Cronbach’s alpha for this scale was 0.78.

#### 2.2.3. Patient Health Questionnaire—9 Items (PHQ-9)

The PHQ-9 was used to assess the severity of depressive symptoms. It consists of nine items and is scored using a four-point Likert scale, with higher scores indicating greater depression severity [23]. The Cronbach’s alpha for the PHQ-9 was 0.87 in this study.

#### 2.2.4. Rosenberg Self-Esteem Scale (RSES)

The RSES was used to assess the self-esteem levels of the subjects in this study. It consists of ten items and is evaluated using a four-point Likert scale, with higher scores indicating greater self-esteem [24]. In this study, the Cronbach’s alpha for the RSES was 0.82.

### 2.3. Quality Control

Before the survey began, the principal investigator coordinated with the drug rehabilitation center to finalize the schedule and location and developed a detailed survey plan. Investigators received uniform training to minimize bias. During the survey’s implementation, investigators explained its purpose and content to participants while distributing the questionnaires. The survey was conducted centrally, enabling participants to independently and anonymously complete the questionnaires according to the instructions. After completing the questionnaires, participants returned them to investigators on-site. The investigators checked for missed or misunderstood answers and provided immediate feedback, asking participants to make necessary corrections or additions. Once the survey was completed, investigators conducted a final check of the questionnaires to eliminate invalid responses, such as patterned answers, before coding. Two data entry clerks entered each questionnaire into a standardized database for double entry.

### 2.4. Statistical Analysis

A mediation model was constructed based on the proposed hypotheses, with parameters estimated using the maximum likelihood method. Model fit was evaluated using the *χ*^2^/*df*, Comparative Fit Index (CFI), Non-Normed Fit Index (NNFI), Standardized Root Mean Square Residual (SRMR), and Root Mean Square Error of Approximation (RMSEA). Acceptable model fit criteria included [25] *χ*^2^/*df* < 3.0; CFI and NNFI > 0.90; and SRMR and RMSEA < 0.08. The bootstrap method, using 5000 samples, was used to test the mediation effect and estimate the confidence interval, with a significance level set at two-sided α = 0.05. A factor algorithm [26] was applied to parcel the AMIS, RSES, and PHQ-9 items, improving model efficiency and reducing parameter estimation bias. A multi-group analysis was performed to test the measurement invariance across patterns of illicit drug use (primary illicit drug of use and years of illicit drug use). When comparing different types of primary drug use, the ketamine users were excluded from the multi-group analysis due to the insufficient sample size (only six participants used ketamine).

## 3. Results

### 3.1. Characteristics of the Participants

The characteristics of the participants are shown in Table 1. The participants had a mean age of (39.87 ± 9.50) years. The average duration of illicit drug use was (10.50 ± 7.34) years.

### 3.2. Descriptive Statistics and Correlations

Addiction memory was significantly positively correlated with depression (*r* = 0.310, *p* < 0.01), while family functioning and self-esteem were significantly negatively correlated with depression (*r* = −0.174, *p* < 0.01). Additionally, addiction memory was significantly negatively correlated with self-esteem (*r* = −0.256, *p* < 0.01), and family functioning was significantly positively correlated with self-esteem (*r* = 0.151, *p* < 0.01). The means, standard deviations, and correlation coefficients are shown in Table 2.

### 3.3. Common Method Bias Test

The Harman single-factor method was used to test for common method bias. Factor analysis was conducted on all items, revealing six factors with eigenvalues greater than 1. The first factor explained 23.81% of the total variance, falling below the 50% threshold. Thus, this study does not suffer from serious common method bias.

### 3.4. Mediation Analysis

**Figure 1:**
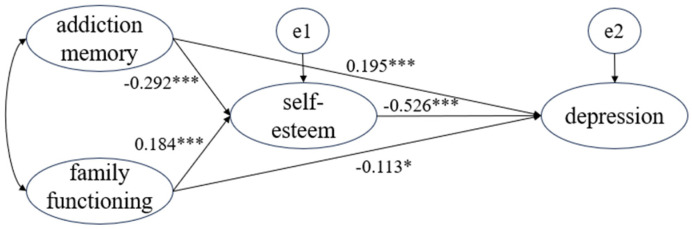
Mediation model of self-esteem. Notes: * *p* < 0.05; *** *p* < 0.001.

The model fit results showed an *χ*^2^/*df* = 2.248, a CFI = 0.956, an NNFI = 0.966, an RMSEA = 0.046, and an SRMR = 0.040, indicating a good fit with respect to the sample data. As shown in Figure 1, all path coefficients were statistically significant (*p* < 0.05). Addiction memory significantly positively predicted depression (*β* = 0.195, *p* < 0.001), while family functioning significantly negatively predicted depression (*β* = −0.113, *p* = 0.008).

As shown in Table 3, the total effect of addiction memory on depression was equal to 0.349, with a mediating effect of self-esteem amounting to 0.154 (95% *CI*: 0.106 to 0.209), accounting for 44.13% of the total effect. The direct effect of family functioning on depression amounted to −0.113, while the mediating effect of self-esteem was equal to −0.097 (95% *CI*: −0.154 to −0.047), accounting for 46.19% of the total effect.

### 3.5. Multi-Group Analysis

As shown in Table 4, all the model fit indexes met the acceptable criteria. When constraining all the parameters so that they would be equal across groups, there was no significant difference between the unconstrained and constrained models (across primary illicit drug of use: Δ*χ*^2^ (12) = 11.920, *p* = 0.452; across years of illicit drug use: Δ*χ*^2^ (12) = 20.322, *p* = 0.612), indicating measurement invariance across the groups of different patterns of illicit drug use.

## 4. Discussion

This study’s aim was to develop a mediation model to explore the association between addiction memory, family functioning, and depressive symptoms among illicit drug users, focusing on the mediating role of self-esteem. This research is the first to investigate the direct effects of addiction memory on depressive symptoms and the underlying psychological mechanisms. The findings revealed that both addiction memory and family functioning independently relate to depressive symptoms. Additionally, self-esteem was identified as a crucial mediating variable that significantly explains how addiction memory and family functioning affect depressive symptoms. The results of the multi-group analysis suggest measurement invariance across groups of different patterns of substance use, indicating that the present mediation model can be reliably used to demonstrate a common mechanism among different illicit drug users.

Addiction memory directly influences depressive states and indirectly exacerbates symptoms through self-esteem, which accounts for 44.13% of the total effect. The formation and retrieval of addiction memory require selective attention. Research indicates that substance abusers often exhibit an attentional bias toward drug-related stimuli. This attentional bias intensifies cravings for substances and significantly increases the risk of relapse [27]. Persistent substance abuse strengthens addiction memory and leads to an imbalance in brain function. This leads to an abnormal increase in signal activity within the neural reward circuitry, particularly in the striatal–limbic network. Concurrently, signaling decreases in the cognitive control circuitry, including executive control and attentional networks [28]. These changes impede cognitive control processes, induce cognitive dysfunction, and hinder substance abusers’ ability to self-regulate healthy behaviors, ultimately affecting their self-esteem.

According to memory reconsolidation theory, the basolateral amygdala is essential for reconsolidating conditioned cue memories and regulating emotions. Exposure to drug-related cues activates the amygdala, facilitating memory reconsolidation and intensifying negative emotions [29]. Therefore, inhibiting amygdala-mediated signaling pathways is crucial for reducing addiction memory intensity and alleviating depressive symptoms. Strengthening cognitive control circuits can help restore cognitive abilities and improve self-esteem. This goal can be achieved through effective interventions, such as cognitive-behavioral therapy.

This study found that family functioning directly affects depression and significantly influences it indirectly through self-esteem, with the mediating effect accounting for 46.19% of the total effect. This finding highlights the essential role of family functioning in alleviating depressive symptoms among illicit drug users by enhancing self-esteem. Effective family functioning promotes positive emotions and psychological well-being among family members, helping individuals establish constructive values [30]. Our study’s findings align with previous research that identified family functioning as a positive predictor of self-esteem [31].

Conversely, substance abusers from dysfunctional families often lack communication and emotional support. This results in diminished self-worth and negative self-evaluations, ultimately reducing their self-esteem. Self-esteem, an important predictor of depressive symptoms, has been shown to negatively correlate with these symptoms in several studies [32,33]. Individuals with high self-esteem tend to have positive self-assessments and realistic self-perceptions. They exhibit emotional stability, optimism, strong problem-solving abilities, and confidence. Equipped with strong psychological regulation and self-control [34], they can effectively self-regulate and resist depressive emotions and substance temptations. In contrast, individuals with low self-esteem have negative self-worth perceptions, dwell on past failures, lack confidence when facing challenges, and often hesitate to seek help. This exacerbates negative emotions and triggers depressive symptoms. Therefore, enhancing self-esteem is an effective strategy for alleviating depressive symptoms in substance abusers. Various therapeutic approaches, including mindfulness interventions [35], cognitive behavioral therapy [36], the Satir therapeutic model [37], and working memory training [38], effectively prevent drug relapse. This emphasizes the importance of bolstering self-esteem as an adjunct treatment for alleviating depressive symptoms in substance abusers.

Although our findings suggest that better family dynamics are associated with lower levels of depressive symptoms, it is important to consider whether this relationship is specific to illicit drug users or reflects a broader pattern observed in the general population. Previous studies have demonstrated that family functioning significantly influences mental health in both clinical and non-clinical populations [39]. For instance, strong familial bonds have been shown to buffer against stress and reduce depression in non-substance-using groups. However, families of illicit drug users often experience unique stressors, such as stigma, financial strain, and interpersonal conflict, which may exacerbate the impact of poor family functioning on mental health [40]. These distinct challenges could amplify the observed relationship in this population. In the future, researchers should conduct comparative analyses between illicit drug users and non-users to determine the extent to which the observed relationship is population-specific. Such studies could help disentangle the influence of substance-related familial dynamics from more general family functioning effects on depression. Moreover, longitudinal research examining changes in family dynamics and their impact on depression following interventions targeting illicit drug use could provide deeper insights into this relationship.

## 5. Conclusions

In summary, this study demonstrates a positive relationship between addiction memory and depression and a negative relationship between family functioning and depression. Furthermore, it emphasizes the significant role of self-esteem as a mediator in the relationships between addiction memory, family functioning, and depression. Based on these findings, targeted interventions that enhance family functioning and reduce the effects of addiction memory may effectively improve self-esteem among illicit drug users and alleviate their depressive symptoms.

### Limitations

This study has several limitations. First, the use of a cross-sectional design limits our ability to infer causal relationships between addiction memory, family functioning, self-esteem, and depression. Therefore, the findings should be interpreted cautiously and not be directly used as evidence for causal links. Second, the sample is limited to illicit drug users in Central China. Although the measurement invariance indicates that the mediation model reflects a shared mechanism applicable to various types of illicit drug users, it should be noted that the participants in this study primarily used methamphetamine and heroin, while other substances such as marijuana and cocaine were excluded, which may constrain the generalizability and applicability of the results. Future research should expand the sample to include diverse regions and types of substance users, thereby enhancing the representativeness of the findings. Third, this study primarily examines how addiction memory, family functioning, and self-esteem influence depressive symptoms. However, the etiology of depression in substance users is complex and involves factors such as treatment readiness, craving, social support, and coping. Also, given the primary aim of our research, detailed information on participants’ trauma histories and comorbid mental illnesses was not collected in this study, which presents a limitation in terms of being able to fully understand the complex mechanisms underlying the relationships. Although we employed validated instruments and robust methodologies to ensure the reliability and validity of our findings within the chosen framework, developing a comprehensive model in future studies that incorporate various variables would provide a complete understanding and shed light on the complex pathways that lead to depression among illicit drug users.

## Figures and Tables

**Table 1:** Characteristics of the participants.

Characteristics		*N*	%
Gender	Male	296	50.08
	Female	295	49.92
Age	<40	292	49.41
	≥40	299	50.59
Education	Primary school or below	77	13.03
	Junior high school	304	51.44
	Senior high school	162	27.41
	Junior college or above	48	8.12
Marital status	Single	193	30.96
	Married/cohabitating	188	31.81
	Divorced/widowed	439	74.28
Received addiction treatment before admission	Not ever	163	27.58
	More than once	428	72.42
Primary illicit drug of use	Methamphetamine	279	47.21
	Heroin	137	23.18
	Ketamine	6	1.02
	Polydrug use	169	28.60
Years of illicit drug use	<10	310	52.45
	≥10	281	47.55

**Table 2:** Descriptive statistics and Pearson correlations between variables.

Variable	Mean	SD	I	II	III	IV
I. Addiction memory	2.79	0.87	1.00			
II. Family functioning	5.86	2.54	−0.008	1.00		
III. Self-esteem	29.36	4.30	−0.256 *	0.151 *	1.00	
IV. Depression	6.73	5.26	0.310 *	−0.174 *	−0.516 *	1.00

Note: * *p* < 0.01; SD = standard deviation.

**Table 3:** Direct effect, indirect effect, and total effect.

Path	Coefficient	95% *CI*	% of the Total Effect
Direct effect			
addiction memory → depression	0.195	0.119~0.268	55.87
family functioning → depression	−0.113	−0.203~−0.025	53.81
Indirect effect			
addiction memory → self-esteem → depression	0.154	0.106~0.209	44.13
family functioning → self-esteem → depression	−0.097	−0.154~−0.047	46.19
Total effect			
addiction memory → depression	0.349	0.268~0.423	100.00
family functioning → depression	−0.210	−0.303~−0.121	100.00

**Table 4:** Multi-group analysis of measurement invariance for patterns of illicit drug use.

Multi-Group Model	*χ* ^2^	*df*	CFI	NNFI	RMSEA	SRMR	Δ*χ*^2^	Δ*df*	*p*
Comparing the primary illicit drug of use									
Unconstrained	513.739	332	0.958	0.954	0.031	0.520			
Constrained weights	525.659	344	0.958	0.956	0.030	0.522	11.920	12	0.452
Comparing the years of illicit drug use									
Unconstrained	344.170	196	0.966	0.959	0.036	0.047			
Constrained weights	364.492	208	0.964	0.959	0.036	0.046	20.322	12	0.612

## Data Availability

The raw data are not publicly available due to ethical restrictions. The data presented in this study are available from the corresponding author upon reasonable request.
